# Characterizing Network Search Algorithms Developed for Dynamic Causal Modeling

**DOI:** 10.3389/fninf.2021.656486

**Published:** 2021-06-10

**Authors:** Sándor Csaba Aranyi, Marianna Nagy, Gábor Opposits, Ervin Berényi, Miklós Emri

**Affiliations:** ^1^Division of Nuclear Medicine and Translational Imaging, Department of Medical Imaging, Faculty of Medicine, University of Debrecen, Debrecen, Hungary; ^2^Division of Radiology and Imaging Science, Department of Medical Imaging, Faculty of Medicine, University of Debrecen, Debrecen, Hungary

**Keywords:** fMRI, dynamic causal modeling, search algorithm, network topology, model-space

## Abstract

Dynamic causal modeling (DCM) is a widely used tool to estimate the effective connectivity of specified models of a brain network. Finding the model explaining measured data is one of the most important outstanding problems in Bayesian modeling. Using heuristic model search algorithms enables us to find an optimal model without having to define a model set a priori. However, the development of such methods is cumbersome in the case of large model-spaces. We aimed to utilize commonly used graph theoretical search algorithms for DCM to create a framework for characterizing them, and to investigate relevance of such methods for single-subject and group-level studies. Because of the enormous computational demand of DCM calculations, we separated the model estimation procedure from the search algorithm by providing a database containing the parameters of all models in a full model-space. For test data a publicly available fMRI dataset of 60 subjects was used. First, we reimplemented the deterministic bilinear DCM algorithm in the ReDCM R package, increasing computational speed during model estimation. Then, three network search algorithms have been adapted for DCM, and we demonstrated how modifications to these methods, based on DCM posterior parameter estimates, can enhance search performance. Comparison of the results are based on model evidence, structural similarities and the number of model estimations needed during search. An analytical approach using Bayesian model reduction (BMR) for efficient network discovery is already available for DCM. Comparing model search methods we found that topological algorithms often outperform analytical methods for single-subject analysis and achieve similar results for recovering common network properties of the winning model family, or set of models, obtained by multi-subject family-wise analysis. However, network search methods show their limitations in higher level statistical analysis of parametric empirical Bayes. Optimizing such linear modeling schemes the BMR methods are still considered the recommended approach. We envision the freely available database of estimated model-spaces to help further studies of the DCM model-space, and the ReDCM package to be a useful contribution for Bayesian inference within and beyond the field of neuroscience.

## 1. Introduction

In recent years of neuroscience, increasing attention is drawn toward brain connectivity studies. Non-invasive techniques, like functional magnetic resonance imaging (fMRI) and electroencephalography (EEG), have gained popularity for this purpose. Dynamic causal modeling (DCM) is a continuously developing Bayesian framework for estimating effective neuronal connectivity between brain regions. It models neuronal signal alteration underlying the fMRI or EEG data for predicting network connectivity, their modulations and the effects of experimental inputs, while physiological parameters of the measured data are also accounted for. Initially, DCM was a hypothesis-driven method, useful to compare a small number of models to test neurobiologically relevant questions (Friston et al., 2003), and using Bayesian model selection to decide which model has the highest evidence (Penny et al., 2004). However, numerous studies focus on exploring a systematically built model-space to find the best fitting model for the data, and to draw an inference from it (Pool et al., 2014; Warren et al., 2019). More recent DCM development enables the comparison of model families of hundreds, or thousands of models along common network properties, and inferencing the parameters of the averaged model of the winning family using Bayesian model comparison and subsequent averaging (Penny et al., 2010). For group analysis the currently recommended standard procedure involves the model inversion of a fully connected DCM model and compute parametric empirical Bayes (PEB) over subjects (Friston et al., 2016). Then, we can test our hypotheses on model commonalities and differences by estimating posteriors of any nested model with a method called Bayesian model reduction (BMR), or perform an automatic search among all nested models to discover connections that most likely contribute to the final model evidence (Zeidman et al., 2019b).

Methods for searching for the model that most likely explains the measured data are also known as structure learning, which is one of the most important outstanding problems in Bayesian modeling. However, discovering large model-spaces is not a trivial task, considering that the number of alternative models grows exponentially with the number of network nodes and external effects. For this reason searching for an optimal solution is less advised in current research. A discovery method for causal networks have already been developed, in the Bayesian framework, to perform *post-hoc* model selection. This method refers to the greedy search to find parameters to remove from the pre-estimated fully connected model, which do not contribute to the final model evidence (Friston and Penny, 2011). This procedure also exploits the efficiency of BMR. With this technique it is only necessary to invert the full model and then estimate any nested models in milliseconds, which is useful when estimating large number of models (Friston et al., 2016). Nonetheless, the standard approach to estimate each model separately still remains relevant, because it is still unclear whether BMR remains robust to nonlinearities, such as the hemodynamic forward model DCM uses (Buxton et al., 1998). Currently, searching methods to find the optimal model structure through fully inverting DCM models have not yet been thoroughly investigated for DCM.

A search algorithm aims to find an optimal solution within the boundaries of a search space that meets or approximates predefined criteria. An iterative search algorithm may start with an initial structure within the search space (e.g., an empty or a fully connected network). Then, we construct topological alternatives to this network by adding or subtracting edges between network nodes. Finally, we evaluate the set of alternatives based on some approximation of the model evidence, for example, Akaike Information Criterion (AIC) or Bayesian Information Criterion (BIC) to select the best ones for the next iteration. In DCM variational free-energy generally gives a better approximation of the log evidence, and is preferred over AIC and BIC (Penny, 2012). For Bayesian networks, many procedures exist for the data-driven discovery of network graph structures (Smith et al., 2011; Mumford and Ramsey, 2014). These include simple greedy equivalence search (GES) methods (Ramsey et al., 2011), and more complex multi-level algorithms combining greedy search with simulated annealing (Adabor et al., 2015).

In the case of DCM, every model search method attempts to find the model with the highest evidence based on fMRI data. As DCM models can be represented as graphs, some commonly used heuristic network optimization algorithms can easily be adapted to search through the DCM model-space. The computational difficulties limit the possibilities to develop searching methods for DCM. In the literature, only a few optimization methods are available to search for the best fitting model. Pyka et al. (2011) investigated the effectiveness of genetic algorithms (GA) compared to a fully randomized search. They demonstrated that GA found better DCM models by estimating fewer models than brute-force methods.

In DCM, one can choose from multiple possibilities for network discovery. On the subject-level the most straightforward path to follow is to directly invert each model alternatives along the search path individually using the variational Laplace (VL) algorithm (Friston et al., 2007). This method is the slowest and it is possible for different models to fall into different local minima during the estimation procedure. Alternatively, one can apply Bayesian model reduction on any DCM in relation to the fully connected model. This method also allows for using *post-hoc* model selection to find an optimal solution in a more analytic approach. The advantage of BMR is that it assumes that all models are evaluated around the same minima of free-energy. However, the approximation of model evidence is not known exactly for DCMs, as BMR assumes that the reduced posterior parameter distributions are Gaussian, which might not be appropriate due to nonlinearities in the model (Friston et al., 2016). Lastly, model search methods can be performed on the group-level among nested PEB models.

In practice searching through a large model-space assumes that each model is potentially equally likely a priori, while some models are usually more plausible than others, which is neglected during Bayesian model comparison. Furthermore, the prior probability density in DCM are composed to decrease the risk of overfitting the data. However, in large model-spaces it is possible to find a setting of priors that allows to fit the data, possibly overfitting it. Finally, interpretation of results of automatic searching methods without any hypothesis about the model is difficult, and hardly reproducible with different subjects or input data. For these reasons it is practical to form model families (of hundreds or thousands of models), each corresponding to a hypothesis about the underlying network, and assign each model alternative to one of these families. In this setup model search algorithms may prove useful if they can recover properties of the winning family.

Considering the topological complexity of DCM models and the underlying neurobiological modeling, as well, the high computational demand of DCM still limits the efficiency to explore large model-spaces. The algorithm for model inversion, used by a variational Laplace scheme to estimate the physiological and connection parameters of the causal model, is computationally costly. This is especially true for DCM for fMRI, because they integrate the neuronal and the hemodynamic states of each region, as well. As an obvious solution to speeding up DCM by graphical processing units, GPU-enhanced calculations was accomplished previously for fMRI (Aponte et al., 2016) as well as event-related potentials (Wang et al., 2013). For our computations, we decided to follow the procedures of the original algorithm. We introduce a complete reimplementation of DCM facilitating efficient computing libraries to gain speed. We refer to this version of the software as ReDCM in the following.

In this study, we aimed to create a computational framework for developing and characterizing different DCM-adapted model search methods, and investigate their uses in subject- and group-level scenarios. To focus on different searching methods we finessed the computational burden of DCM model inversion by pre-computing model evidence of every possible model in the model-space generated from the fMRI dataset used in Zeidman et al. (2019a). This enables to compare an arbitrary amount of DCM models without fitting them on the fly. Looking up estimation results from a pre-computed database helps efficient testing and development of search procedures. Furthermore, knowing the best fitting model of the model-space, we can easily measure the performance of the investigated methods. We adapted three model search algorithms for DCM that is available for network science. These are the above mentioned GES and GA algorithms, and a variant of the greedy method based on Hamming-distance of model structures. We refer to this algorithm as GHD from now on. We also looked into the possibilities to improve topological model search procedures by taking into account the DCM parameter estimates from previously reached models during the search. Finally, we characterized and compared the efficiency of these algorithms applied in relation to single subject analysis, family-wise inference and group-level PEB modeling.

## 2. Materials and Methods

### 2.1. Mathematical Background of Dynamic Causal Modeling

We reviewed the DCM mathematics from the point of view of full model-space generation and reimplementation. For the generation of all DCM models, we examined how the DCM implementation handles the topology of internal and external interactions of the neuronal networks. The mathematical background of this topology can be revealed in the neuronal state equation of DCM.

DCM for fMRI models neural interactions between brain regions of a specified network. At any time point, the state of neuronal activity of each region depends on its neural state *x* at the previous time point and can be perturbed by experimentally driven stimuli *u*. In DCM the temporal change of the neuronal state vector is modeled using a bilinear Taylor series approximation, truncated to its linear terms (Stephan et al., 2008). This scheme can model any nonlinear function *f*(*x, u*) around the system's resting point. These time-dependent dynamics can be expressed as the differential equation below:

(1) $$\begin{array}{l} \text{f}\left(\text{x},\text{u}\right)=\frac{dx}{dt}\approx f\left(0,0\right)+\underset{A}{\underset{︸}{\frac{\partial f}{\partial x}}}x+\underset{B}{\underset{︸}{\frac{\partial^{2}f}{\partial x\partial u}}}xu+\underset{C}{\underset{︸}{\frac{\partial f}{\partial u}}}u, \end{array}$$

where the network dynamics are computed around the *f*(0, 0) point. Jacobian matrices *A*, *B* and *C* are parameters of the three different kinds of neuronal interactions modeled by DCM: the endogenous connectivity of the network, the modulatory effects of external input on the connections and the direct or driving effects of the stimuli (input) on the regions, respectively. These parameters define the model topology describing the inter-regional connections, and the external stimuli induced regional activity alterations and regional interaction modulations.

The temporal neuronal activation needs to be combined with a modality-specific forward model to explain regional BOLD (blood oxygen level dependent) fMRI responses. DCM for fMRI uses a hemodynamic model based on the Balloon-Windkessel (or simply Balloon) model (Buxton et al., 1998), that is adapted and extended for DCM (Friston et al., 2000). In this model, the hemodynamic states are a function only of the neuronal state of the regions and represent the volume and deoxyhemoglobin content of the flowing blood. The full forward model of neuronal and hemodynamic state equations is used to predict the BOLD signal *h*(*u*, Θ) of network regions, where Θ are the parameters of the neuronal and the hemodynamic models. Because of the nonlinearities in the Balloon-model, the differential state equations need to be integrated numerically that can be extremely demanding on computational power.

For parameter estimation, a Bayesian framework is used. An iterative variational Laplace (VL) algorithm optimizes the maximum a posteriori (MAP) estimate of the free model parameters (Friston et al., 2007). By integrating out the dependencies between parameters we obtain model evidence, which can be used for model selection or comparison. In DCM, variational free-energy (Fe) is used to approximate model evidence. The free-energy balances between the fit of the model to the data and complexity, like the number of free parameters in the model (Stephan et al., 2007). Hence, Fe is useful for comparing models while eliminating the effects of overfitting the data on model evidence.

### 2.2. Optimized DCM Implementation

Considering the computational demands of DCM, originally available as part of the Statistical Parametric Mapping (SPM, http://www.fil.ion.ucl.ac.uk/spm/) Matlab toolbox, evaluating large amounts of models requires unmanageable processor time. To overcome this limitation we reimplemented the deterministic bilinear DCM12 algorithm (build v6225) in the R package ReDCM. (DCM12 refers to the actual implementation of the DCM algorithm in the given SPM version). The whole estimation procedure of variational Laplace and the DCM forward models for fMRI (neural and hemodynamic states) is available in ReDCM. The R programming language provided us a feasible environment to implement the Bayesian estimation framework, data analysis methods and model search algorithms as well. Besides DCM for fMRI, ReDCM also implements BOLD signal simulation, Bayesian model selection using fixed effects statistics or random effects with Gibbs-sampling (Penny et al., 2010), and a separate tool to observe hemodynamic response function (HRF) for a set of Balloon-model parameters.

Previous studies have identified the computation bottlenecks of DCM (Aponte et al., 2016), namely the integration of differential state equations describing temporal neuronal and hemodynamic changes, which needs to be optimized. These parts of the code were ported into C language using the GNU Scientific Library (GSL) (Galassi et al., 2006) for efficient matrix calculations. Four virtual machines with 48 CPU cores each were acquired in Microsoft's Azure cloud platform (Copeland et al., 2015) to utilize high-performance computing facilities for estimating multiple DCM models simultaneously.

We measured the performance of ReDCM and DCM12 without any parallelization techniques to quantify the computing efficiency we gained. For this analysis we generated synthetic BOLD-signal data of varying scan length between 200 and 1,200 time points and DCM models with different model sizes, containing 3, 5, and 7 interconnected regions of interest. We show the average runtime of iterative VL cycles, measured with both implementations, estimating parameters of each synthetic model. Each model had two external stimulating effect to drive regional state dynamics. For the BOLD time-series simulation we used the ReDCM implementation of appropriate functions from SPM.

### 2.3. Model-Space Generation for the Semantic Decision Task

In the case of a neural network, the number of all mathematically possible models (i.e., the cardinality of the full model-space, *N*_*ms*_) increases hyper-exponentially, depending on the number of regions and experimental inputs. As the parameter priors can be expressed as binary variables (connected or not connected), the number of possible bilinear models can be computed with a simple expression:

(2) $$\begin{array}{l} log_{2}N_{ms}=\left(n^{2}-n\right)+i*\left(n^{2}\right)+i*n, \end{array}$$

where *n* is the number of regions and i is the number of input functions. The first additive term describes the endogenous network connectivity (*A*), the second adds the number of possible modulatory effects of inputs (*B*) and the third counts the direct effects on each region (*C*). We subtracted the number of regions from terms related to the A matrix, because self-connections always represent self-inhibitory effects which need to be estimated.

For test data, we used the fMRI BOLD dataset freely available as supplementary data from Zeidman et al. (2019a), which is used to investigate laterality of semantic processing before (Seghier et al., 2011). This consists of the same specified DCM model for 60 subjects, from which we used the first 10 for individual level computations, and data for group-wise PEB model. The experimental design involves three conditions: “Pictures,” “Words” includes onset for the corresponding semantic decision trials and “Task” includes all trials. The network architecture consists of four regions in the frontal cortex, responding to language processing: left ventral (lvF), left dorsal (ldF), right ventral (rvF), and right dorsal (rdF). For keeping consistency with previous work, and for computational reasons, we constrained the full model-space by fixing the *C* matrix so that only the “Task” condition is used as driving inputs for each region, while “Pictures” and “Words” are used for modulatory effects. In accordance with Zeidman et al. (2019a), Figure 1 shows the model considered as fully connected in this experiment. Keeping the constraints described here in mind this semantic decision network induces a model-space of 65,536 possible nested models to consider for each of the 10 subjects.

**Figure 1:**
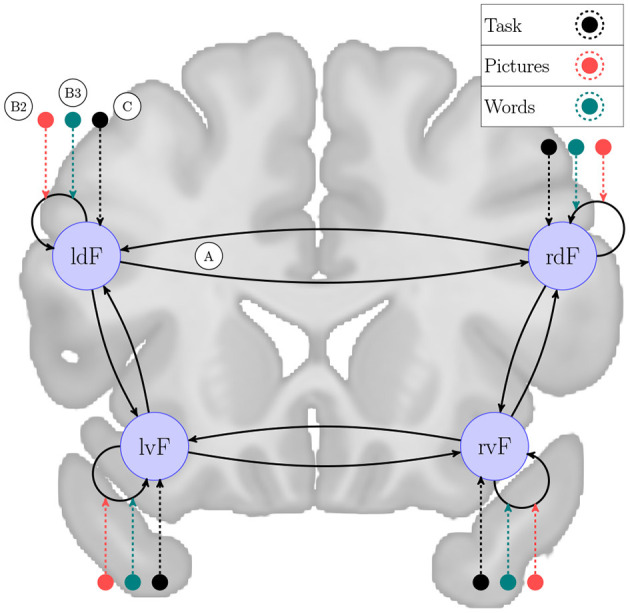
Network scheme for semantic decision task. A DCM model example to explain brain functions during semantic processing of words and pictures, originally investigated by /Seghier 2011/. The experiment consists of four frontal brain regions: left ventral (lvF), left dorsal (ldF), right ventral (rvF) and right dorsal (rdF). This network is examined in a task designed to involve three conditions: “Pictures” and “Words” includes onsets for the corresponding semantic decision trials and “Task” includes all trials. We generated a model-space of 65,536 models with every possible combination of endogenous connectivity matrix A, along with their experimental modulation, denoted by B1, B2, and B3. We fixed the direct effects, described by matrix C, to “Task” driving each region as experimental input. On the figure the network nodes are overlain on a coronal section of the brain captured from the average brain template of the Montreal Neurological Institute (Grabner et al., 2006).

Group-level analysis is designed to inference on different lateral responses between left and right regions during the processing the semantic content of familiar words, quantified by the “Laterality Index” (LI). In the group-level PEB analysis several effects are modeled similarly to a general linear modeling scheme. Most importantly the commonalities, or main effect among all subjects and the LI to model subject variability. Handedness, gender and age are also included to capture variance no interest. Detailed description of the experiment is found in Seghier et al. (2011).

Taking advantage of the ReDCM implementation and high performance computing, we estimated all models of the constrained model-space, and organized the estimated parameters of all models into an easy to handle data table. Description of this database is provided in Table 1. Alternatively, we also estimated the model-space using BMR. To examine both statistical and structural attributes of the model-space, we expanded this table with the DCM models' free-energy (Fe), and their Hamming-distance (Hd) relative to the model with the highest evidence. If we represent *A*, *B*, and *C* matrices as directed cyclic graph structures, we can describe the topological difference of any two models by their Hamming-distance. The Hamming-distance of two models is defined as the number of different graph edges that describe their connectivity.

**Table 1:** Model-space database description.

**Name**	**Abbreviation**	**Description**
Model identifier	ID	Unique numerical identifier of a model.
Free-energy	Fe	Estimated free-energy of a model.
Free-energy difference	dFe	Difference in free-energy relative to the best fitting model.
Hamming-distance	Hd	Hamming-distance of a model to the best fitting model.
Vectorized model priors	Bitvector	A bitvector representing the model structure topologically, constructed from the Vectorized model priors of A, B, and C matrices.
Endogenous connectivity	A	Matrix of endogenous connection parameter estimates.
Modulatory effects	B1, B2, B3	Matrices of connection modulation parameters for each experimental input.
Driving input	C	Direct or driving effect of experimental stimuli.
Connection probability	pA	Matrix of endogenous connection parameter probabilities.
Modulation probability	pB1, pB2, pB3	Matrices of modulation parameter probabilities for each experimental input.
Input probability	pC	Probability of driving effect of experimental stimuli.

*The database of the model-space consists of the parameter estimation results of each DCM model as a computer file that are connected to a summary table by a model identifier. This table collects the basis of model comparison information in statistical (free-energy, connection matrix parameter estimates) and graph theoretical (Hamming-distance, model priors) space*.

### 2.4. Model Search Methods

**Figure 2:**
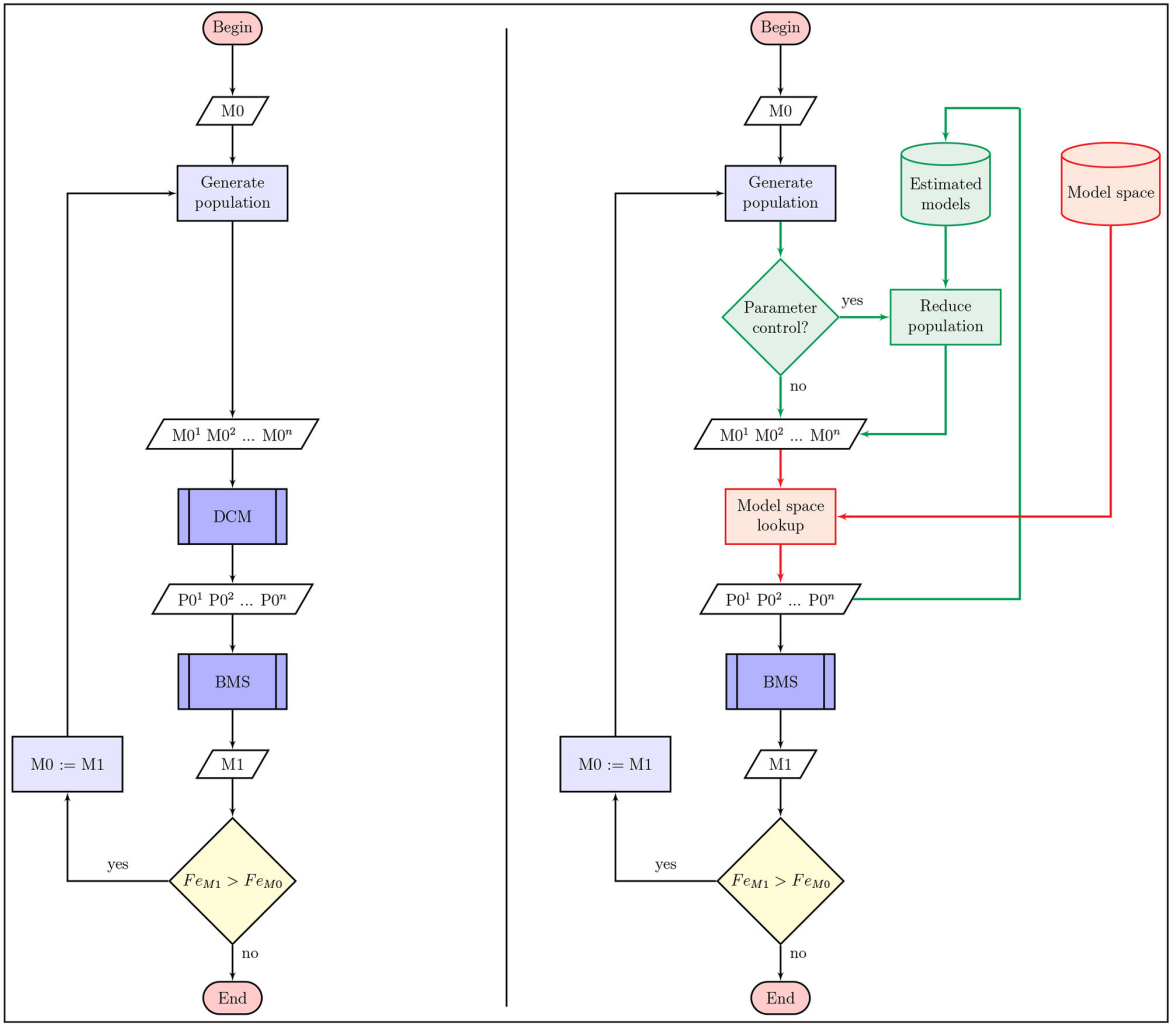
Flowchart of model search algorithms for DCM. The flowchart on the left side panel depicts the most simple schematic of model search methods in a DCM model-space. On the right side we show the changes to the regular procedure that may optimize the algorithm in terms of number of estimated models or search results (green parts), and helps rapid characterization of model search methods using model-space lookup (red parts). In any case the algorithm starts with an initially selected model (or set of models) *M*_0_ that is used to select models $M_{0}^{1},M_{0}^{2},\ldots ,M_{0}^{n}$ with an arbitrary method for DCM computation. Then we can select the best fitting model (or models) *M*_1_ of the selected population with Bayesian model selection, which is used to generate the next set of models to compare in the next iteration. This procedure continues until we cannot find an improved variation of the previously estimated models. As shown on the right side image, posterior parameter estimates of previously reached models $P_{0}^{1},P_{0}^{2},\ldots ,P_{0}^{n}$ can also be used to reduce or manipulate the selected population and improve search efficiency.

Search algorithms aim to find the optimal model with the combination of connectivity parameters that yield the highest evidence of DCM model estimation on the fMRI data. We adapted three different model search algorithms to DCM and characterized their performance. As the connectivity parameters of *A*, *B*, and *C* matrices define the model-space, searching through it is actually about finding the optimal model with the combination of connectivity parameters that yields the highest evidence of DCM model estimation on the fMRI data. The comparison of model search algorithms is based on two main factors: the difference between the estimated Fe of the found model and the best fitting model of the model-space (i.e., how optimal is the result of search), and also the number of models estimated until convergence is reached (i.e., how fast the algorithm converges to the optimum). Based on the applied search method the number of models considered can still be relatively high. However, replacing DCM computations with looking up records from the already estimated model-space, as depicted in Figure 2, makes developing and testing new search algorithms faster.

Another way to improve model search efficiency is to omit models from the current iteration of the algorithm, that differs from previously computed DCM models only in parameters that don't effectively change model evidence based on the Bayesian estimation procedure. Thus, we created optimized versions of the following model search algorithms that skip DCM models that adds or subtracts parameters that are receding or exceeding a definite posterior parameter probability in previously estimated models. Doing this we ensure that connections with high (or low) average probability don't get deleted (or added) by any alternative models. This search space reduction method is outlined with green on Figure 2. The optimization, informed by DCM model estimation, may significantly reduce the number of reached models during a search procedure. However, it needs to be applied with caution as it may introduce possibilities to deflect the search path into a local minima.

#### 2.4.1. Greedy Equivalence Search—GES

Our greedy search algorithm is based on the Greedy Equivalence Search described by Ramsey et al. (2010). It starts off by selecting an initial, randomized dynamic causal model, and every model with one removed connectivity parameter is evaluated with DCM. Then we select the winning model structure with the highest Fe. After that, we estimate each nested model obtained by removing one possible connectivity parameter from the winning model, keeping consistency with model-space restrictions. When we reach a stage when removing the connections do not change the winning model, a backward procedure is started that adds connectivity parameters to the selected model. Forward and backward steps alternate until no model can be found to improve model evidence. The advantage of this method is it always converges at a particular local maximum based on the initial model; however, it cannot leap through them and may never find the best model.

The optimized algorithm, denoted as GES', takes the posterior parameter probabilities into account when generating the set of models to be estimated. Connection parameters can be removed from the model only if their mean posterior probability is below 0.9. With this modification we fix all connections that have a significant impact on model evidence.

#### 2.4.2. Greedy Hamming-Distance Search—GHD

A more general case of GES considers the models as vectors of binary connectivity parameters, rather than graph structures. Similarly, the Hamming-distance based Greedy Search (GHD) starts with a randomized initial model and estimates every model that is at most 1 Hamming-distance far from that model. At the next stage, the model with the highest evidence is selected to repeat the procedure. When no further winning model is found, the algorithm terminates. The idea behind this algorithm is that it is assumed that the models around the best fitting model also have high model evidence (Pyka et al., 2011). Another advantage of this method is that it incorporates forward and backward search at the same time, as well. We applied the same optimization for GHD' that was applied on the GES' algorithm.

#### 2.4.3. Genetic Algorithm—GA

Genetic algorithm is a widely used concept for different optimization and search problems otherwise challenging to solve procedurally. The candidates of the solution (called individuals or chromosomes) are represented as a set of attributes whose combinations describe the individual. These attributes are combined or swapped between individuals in a population of candidates and simulate genetic operations, like crossovers or mutations, to create more viable individuals. In our case, the models are the individuals and its connectivity parameters are the attributes.

The genetic algorithm we use is described in details by Pyka et al. (2011). The genetic code of the models are represented as binary bit vectors of connectivity parameters. First, the procedure selects a random model from the search space and creates three variations of it by randomly changing some bits in the code. Second, using these four individuals, we generate 16 new codes by crossover and mutation genetic operators. Crossover is performed between two chromosomes by swapping a section of the code between two randomly chosen crossover points. A mutation occurs on the new genetic codes with a probability of 50% and changes two to eight randomly selected parameters. If a created model does not satisfy the model-space constraints described above or the code has already been considered during the algorithm, a new model is generated until we have a population of 20 models. Third, all models are estimated by DCM, and Bayesian model selection (Penny et al., 2004) is performed to select the best four models that enter the next iteration of the GA. This particular use of model evidence or free energy for a genetic algorithm provides a nice metaphor for an natural selection as nature's way of performing Bayesian model selection. In other words, there are formulations of evolution in terms of minimizing free energy or maximizing adaptive fitness, where adaptive fitness is simply the marginal likelihood of a phenotype (Campbell, 2016). This procedure stops when no new model is selected in the last three iterations.

Similarly to previously specified search methods, our modified GA' algorithm is also informed by posterior parameter probabilities. Each model that contains connections with average probability *p* < 0.3 is replaced with a new model that is mutated from the population.

### 2.5. Model Search Characteristics

Two routes of individual-level model search were followed. In the first one we performed search among separately estimated DCM models using the adapted algorithms. In this scheme we can compare search efficiency of the GES, GHD, and GA algorithms on the DCM model-space. Another approach involves using BMR to derive model evidence and posterior parameter estimates from the fully connected model. In this BMR model-space the same search methods can be applied. Additionally, *post-hoc* model search results can be compared with topological search methods.

We analyzed each adapted graph-based model search method from the aspects of model fit (Fe), graph structure of the found model (Hd from the best model), and the number of estimated models (N) until the algorithm converges. Note that the GES and GHD procedures are inherently deterministic methods, and always find the same model with the same initialization. However, randomized initialization allows us to measure search performance more accurately. The stochastic methods such as GA, has a different convergence point each run regardless of the initial set of models. Consequently, we derived the efficiency of the implemented search algorithms from 20 consecutive runs for each subject's data, and assessed the model search characteristics by computing the results' mean and standard deviation.

### 2.6. Family-Wise Inference

The model families should be created to correspond to hypotheses about network structure attributes that are of interest by the experimenter. In case of BMS, both the null hypothesis and alternative hypotheses are compared to each other to make inference from the family that most likely to describe the structure of the network.

In case of our dataset three meaningful separation of the model-space can be made (Zeidman et al., 2019a). In the language related task of semantic processing of shown words and pictures it is more likely that words will have more impact modulating the connections in the network. Also, language processing is considered to dominantly activate regions in the left hemisphere, also with some right side activation, and it might be interesting to see dorsoventral separation of brain function during task. Along these observations three different separation of the model-space can be made: (1) network connectivity is modulated during processing words or pictures or both; (2) connectivity of left side, right side regions, or both sides is modulated; (3) and ventral or dorsal frontal regions are more involved during task or both. The dataset contains 27 base modulation models separated into three equally distributed sets of models for each of the three questions asked. Along these different settings of modulatory effects we assigned every combination of endogenous connectivity parameters to the corresponding model family. This means 2^8^ = 256 models assigned to each base models, as there are eight free parameters found in the *A* matrix. We then performed a random effects analysis (RFX) of family-wise comparison along the group of ten subjects using the same set of models.

Although these families do not cover the entire model-space we can decide for each model that to what extent they may belong to the base models. This is determined by comparing modulatory effects in the found model structure to the modulations defined by the 27 base models. The base model (or models) matching with the highest percentage determines the model family we assign model search results to. When more base models shares the connectivity of the found model, then corresponding families share the model accordingly. It is possible that the found model will show commonalities with more than one model family to some extent. Finally, we summarize model search results for all subjects to determine accuracy to recover family properties.

### 2.7. Search Over Nested PEB Models

For group-level analysis of DCM data the currently recommended procedure is to perform a linear PEB analysis of a fully connected network estimated for all subjects. A common tool to discover network structure on the group-level is based on the same greedy algorithm as *post-hoc* model selection, and uses BMR to evaluate a large number or nested PEB models. As reduced posterior probability can be analytically derived from linear models, BMR is safe to use in conjunction with PEB methods. Thus, using BMR estimated model evidence as decision criterion we compared the implemented topological search methods against the automatic search used in the SPM software. We then show the connectivity parameters for both commonalities and LI that are likely to contribute to the PEB model on the group-level.

## 3. Results

### 3.1. Performance Improvements to Estimate Model-Space

**Figure 3:**
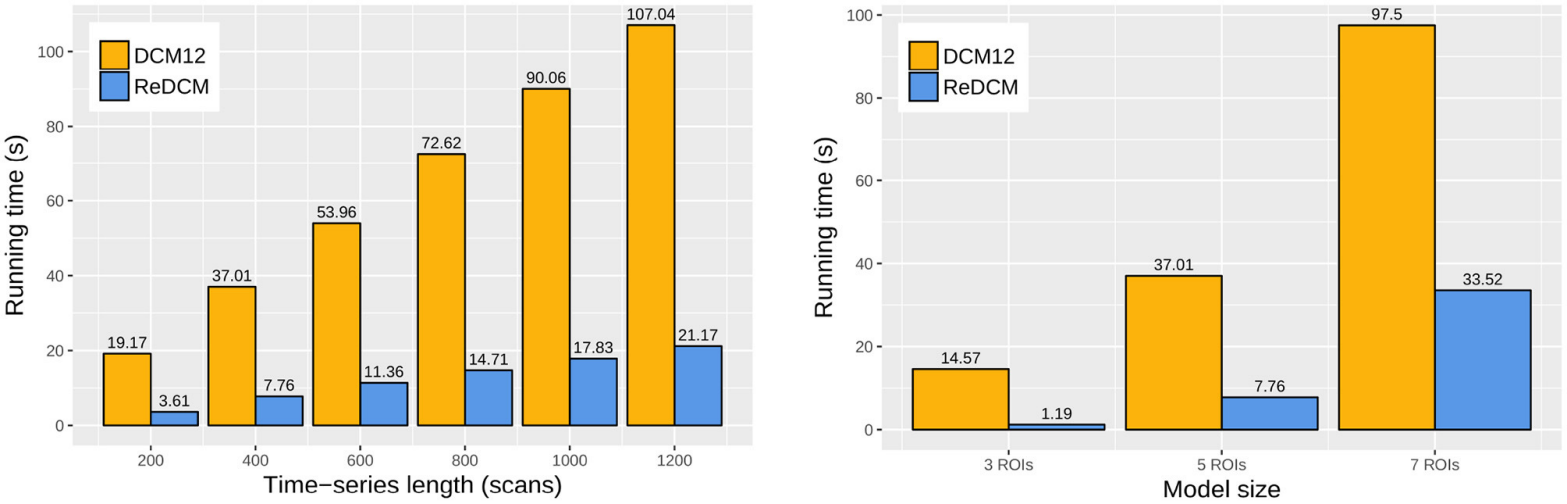
Runtime comparison of DCM12 and ReDCM. We measured the average one-threaded computation runtime of the variational update cycles for both implementations. The comparison was based on varying scan length (200, 400, 600, 800, 1,000, and 1,200 frames) of a DCM model with five regions of interest (ROIs), and different model sizes (3, 5, and 7 ROIs) fitting fMRI data with scan length of 400 frames. There is a linear dependence between scan length and runtime, however, computations are exponentially longer with higher model sizes.

The reimplemented model estimation procedure in ReDCM achieves a significant improvement in computational performance. We compared the variational iteration runtime of ReDCM and DCM12 by estimating the 18 test models by six different scan length (200, 400, 600, 800, 1,000, and 1,200 scans) by three models of varying model size (3, 5, and 7 regions of interest). Figure 3 shows the runtime comparison of every model estimation with model size 5 and length of 400 time points. Computation times of all 18 models are summarized in Table 2. Using ReDCM, without any parallelization or high-performance computing techniques, performance increased by 296–1,078%, depending on size and length.

**Table 2:** Summary of runtime comparison.

	**3 regions**		**5 regions**		**7 regions**	
	**DCM12**	**ReDCM**	**DCM12**	**ReDCM**	**DCM12**	**ReDCM**
200 scans	7.44	0.69	19.17	3.61	53.8	17.19
400 scans	14.57	1.19	37.01	7.76	97.5	33.52
600 scans	21.59	1.57	53.96	11.36	136.37	46.27
800 scans	28.26	1.94	72.62	14.71	189.02	63.35
1,000 scans	34.94	2.34	90.06	17.83	233.77	78.83
1,200 scans	42.48	2.78	107.04	21.17	272.53	91.84

*All 18 synthetic models of different model size and varying scan length were estimated with both DCM12 and ReDCM. This table summarizes the running time of one iteration of the VL algorithm in seconds during parameter estimation*.

As ReDCM is intended to be an exact reimplementation of the DCM algorithm, we did not perform validation using the simulated data. Comparing posterior parameter estimates we found that the average difference between the two versions is lower than 1*10^−4^. Precision differences originate from different low level software libraries used for numerical methods.

### 3.2. Attributes of the Full DCM Model-Space

Running ReDCM on computers 4 * 48 CPU cores, we were able to estimate the full model-space of 10 subjects each containing 65,536 models in 12 days. We organized the estimated parameters of all models into an easy to handle data table. Description of this database is provided in Table 1. Alternatively, we also estimated the model-space using BMR. For investigating the model-space we introduced two attributes: (1) measuring the accuracy by *dFe* = *Fe*0−*Fe*, where *Fe*0 denotes the free-energy of the best model, and (2) the topological distance by *Hd*. Based on the estimated model-space of 10 subjects the *dFe* and *Hd* shows a significant, but moderate correlation with a coefficient of *r* = 0.3(*p* < < 0.001). This is partly in line with the hypothesis of Pyka et al. (2011), that the higher the log evidence difference between models is, the higher the Hamming-distance between them should be on any model-space. This indicates the usefulness of topological model search methods that use the negative free-energy as their fitness function. We also found that in the BMR model-space this correlation between model characteristics is even higher with *r* = 0.45(*p* < < 0.001). The main reason could be that with BMR every model is evaluated around the same minima of free-energy, which is the full model of the model-space.

### 3.3. Subject-Level Characterization of Model Search Algorithms

**Figure 4:**
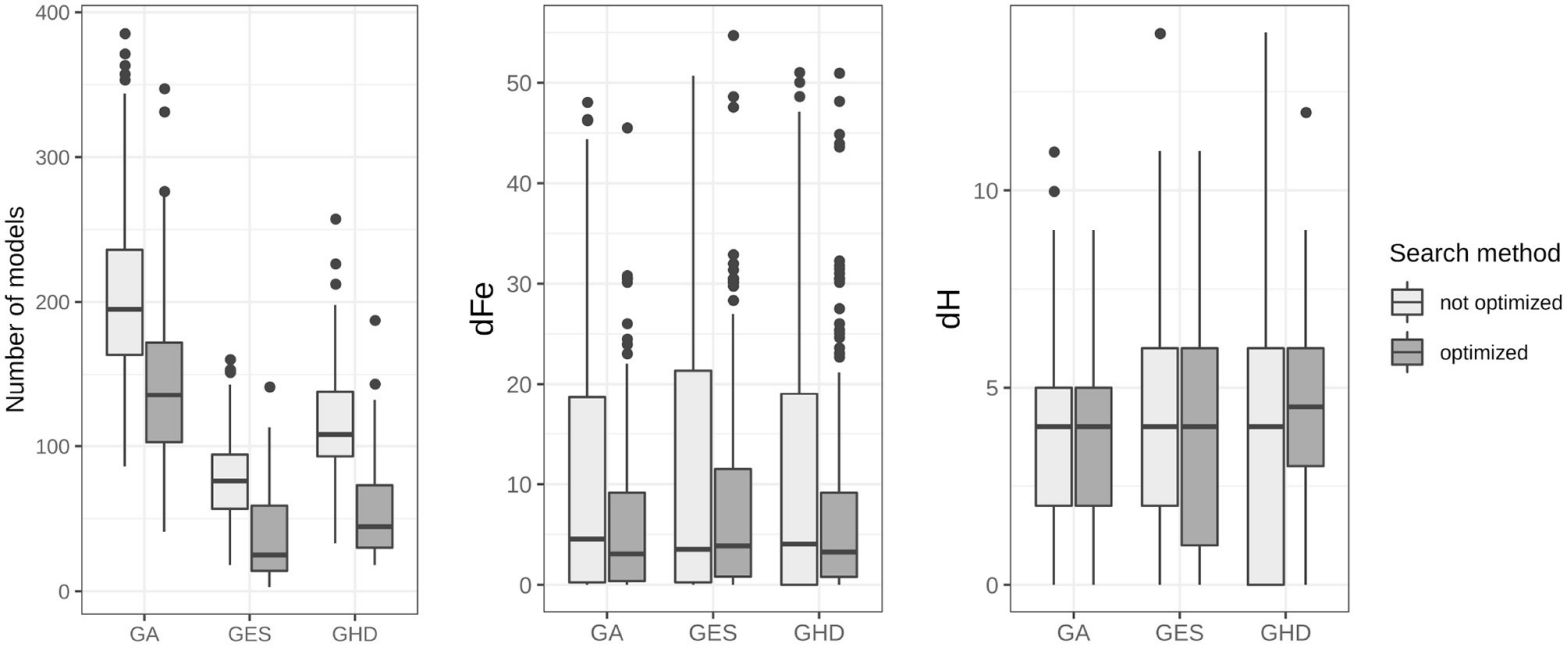
Summary of model search results for topological search algorithms. The accumulated number of estimated models, the dFe and the Hamming-distance of each of the three model search methods are displayed for all 20 runs on each of the 10 subject data. Box-and-whisker diagrams show the median, first and third quartiles of search results with the 95% confidence interval of the median. The improved version of the algorithms needed significantly lower number of models to calculate during search, with *p* < 0.001 for each algorithm.

Results for model search in the DCM model-space are shown in Figure 4. Averaged statistics for the 10 subjects can be found on the left side of Table 3. In our model-space the GA method slightly outperformed the deterministic GES and GHD by finding models having dFe 10.59 relative to the best model in average. However, GA estimates 202 models, roughly twice as much as GES (77) and GHD (118) until the algorithm finishes. Also, the stochastic method tends to find models slightly closer in structure, and having lower Hd (3.71) on average, than the two deterministic approaches (4.28 for GES and 4.07 for GHD).

**Table 3:** Summary of model search results within the model-space estimated by DCM and BMR.

	**DCM**					**BMR**				
**Method**	**dFe**		**Hd**		**N**	**dFe**		**Hd**		**N**
*GES*	13.79	(sd = 24.04)	4.28	(sd = 3.17)	77	**0.24**	(sd = 0.46)	4.30	(sd = 2.19)	81
*GES*′	11.15	(sd = 15.64)	4.23	(sd = 2.77)	36	20.98	(sd = 47.32)	5.43	(sd = 2.64)	30
*GHD*	11.45	(sd = 15.24)	4.07	(sd = 3.37)	118	0.25	(sd = 0.43)	4.35	(sd = 2.30)	129
*GHD*′	8.76	(sd = 11.94)	4.29	(sd = 2.60)	53	7.66	(sd = 26.42)	4.73	(sd = 2.44)	42
*GA*	10.59	(sd = 13.74)	**3.71**	(sd = 2.68)	202	0.74	(sd = 1.38)	4.22	(sd = 2.10)	185
*GA*′	**7.37**	(sd = 10.27)	3.83	(sd = 2.46)	140	5.54	(sd = 19.75)	4.51	(sd = 2.24)	97
*post-hoc*	16.96	(sd = 18.61)	4.80	(sd = 2.30)	NA	0.73	(sd = 0.54)	**2.10**	(sd = 1.66)	NA

*Model search algorithms are characterized based on statistical (Fe) and topological or structural (Hd) properties of the found models and the number of models needed (N) to be estimated until model search converges. Statistics are acquired and averaged for 10 subjects and 20 runs of each method. We included mean dFe, Hd and their standard deviation. Optimized modifications of the base algorithms are denoted as GES', GHD', and GA'. Bold text highlights the best performing method regarding mean dFe and Hd*.

Using the implemented modifications to the original methods we managed to exploit DCM's ability to estimate every connection strength parameter. For each of the three search procedures, the implemented modifications succeed to improve efficiency. Simply fixing parameters based on previously estimated model posteriors the optimized GA' method found the best model multiple times and reached an average of 7.37 dFe. Furthermore, each optimized algorithm need to compute significantly less models until convergence.

**Figure 5:**
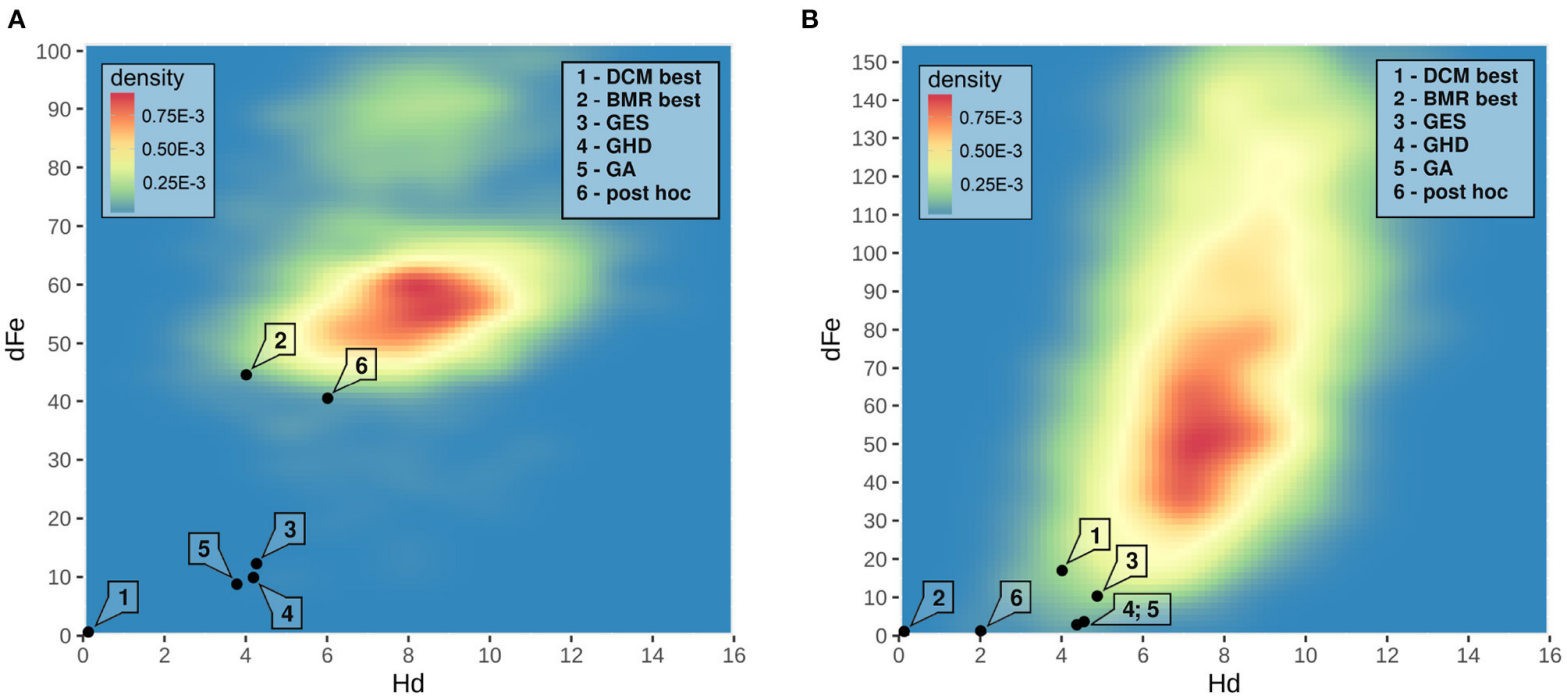
The joint distribution density of dFe and Hd of an example subject. Joint density images reveal any topological structure over the model-space by characterizing the distance between graph structure, as scored with the Hamming-distance, in terms of differences in model evidence or free-energy. The joint density for the model-space estimated by DCM is shown on **(A)**, and the distribution in the BMR space on **(B)**. In cases where there are no relationship between topological structure and model evidence, the joint distribution would look more evenly distributed over Hd in any range of dFe. In this shown example model structure appears to be correlated to model evidence by *r* = 0.33 in the DCM model-space and by *r* = 0.68 in the BMR space. This reflects the assumption of Pyka et al. (2011), being models with higher Fe are also close to the topological structure of the best model. On the (0,0) coordinates the best model of their corresponding space can be found. Model search results are labeled according to their description. As the Bayesian *post-hoc* model selection is not available in the fully estimated DCM model-space, we indicated the model with the same ID on both panels.

Searching through the model-space using BMR allows us to compare the developed methods to the *post-hoc* model search implemented in SPM. Results are shown on the right side of Table 3. Interestingly, GES and GHD methods perform better in the BMR model-space than the other methods, but the optimized algorithms become unreliable. Fixing connectivity parameters in the BMR space with the same criteria used in the DCM space does not improve search efficiency. In terms of Hamming-distance, the *post-hoc* method finds the models closest to the best model in the model-space estimated by BMR. As an example Figure 5 depicts model search results for one subject over the joint distribution of dFe and Hd in both the DCM and the BMR model-space.

### 3.4. Family Inference and Model Search

**Figure 6:**
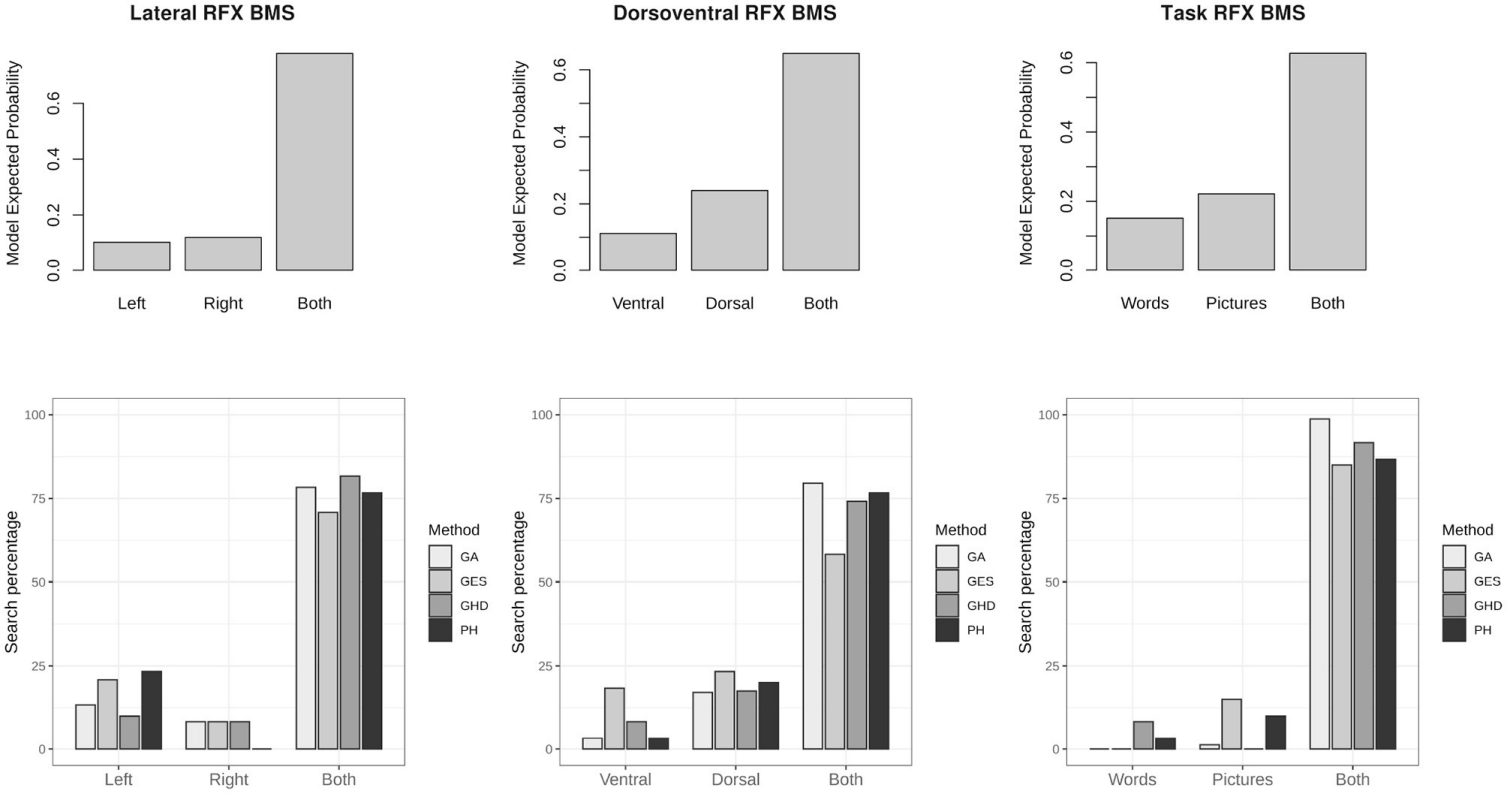
Family-wise inference and family matched by model search methods. The top row shows random effects (RFX) family-wise model selection results over a group of subjects according to laterality, dorsoventral differences and task based differences. The bottom row shows percentage of model search results matching each of the families. The genetic algorithm (GA), the greedy Hamming-distance search (GHD), and *post-hoc* (PH) methods have the highest chance to match properties of the winning model family.

As a step toward group-level analysis, family-level inference is drawn from groups of models over the population. In many cases the models need to be estimated for selecting the winning family. Model search methods may be useful to recover the properties of families. Figure 6 shows the RFX model selection of all three partitioning of the model-space. In each case model search results showed commonalities with the winning family. The GA, GHD, and *post-hoc* model selection algorithms performed similarly between 75 and 99% accuracy to match task-based modulation patterns of the winning families.

### 3.5. Model Search on Population-Level PEB Model

**Figure 7:**
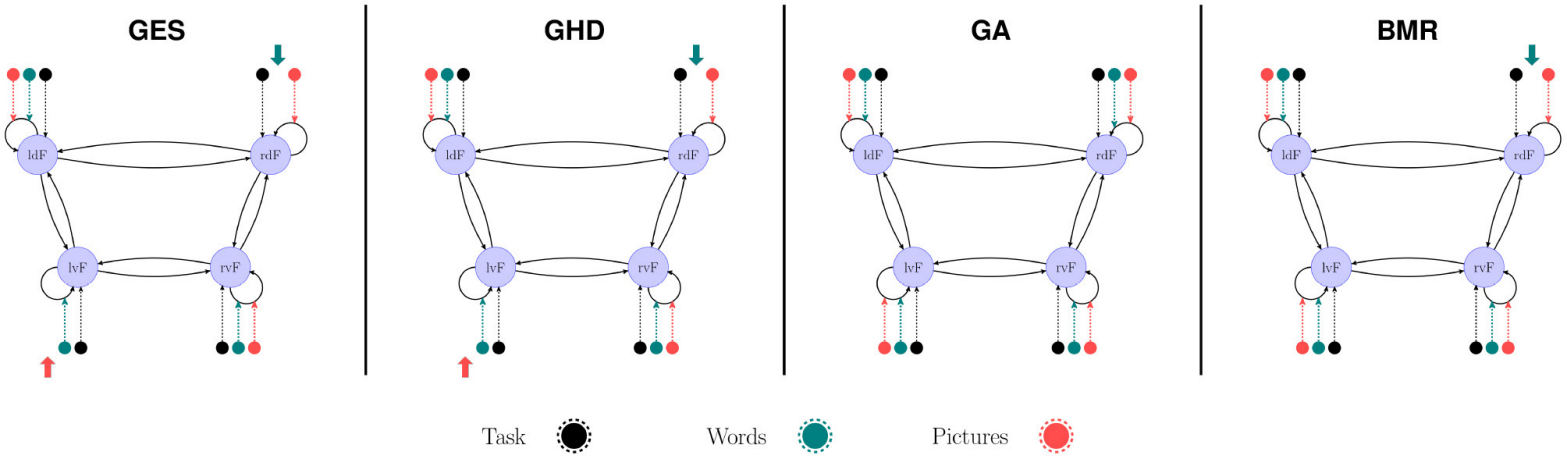
Model search in group-level PEB. The BMR method removed only the modulatory effect of “Words” on the self-inhibition of rdF region. The GES and GHD algorithms also removed the modulatory effect of “Pictures” from lvF self-connection. The GA method found the fully connected model each time regardless of the initial model.

We compared methods for searching among nested PEB models using the automatic greedy method (denoted as BMR in Figure 7) used in SPM and the three topological model search algorithms. In the case of the group-level model we initiated GES and GHD methods with the full model and GA with randomized connectivity. As topological methods are not suitable to search group commonalities (group mean) and LI (differences) simultaneously, we only compared model structure in the group mean effect. We found that BMR removed only the modulatory effect of “Words” task on rdF self-inhibition. The GES and GHD methods also removed this effect along with the effect of “Pictures” on lvF. The GA method returned the full model in each of the 10 performed runs.

## 4. Discussion

Investigating the neuronal interactions between brain regions is encumbered by several technical and methodological challenges. Dynamic causal modeling provides a methodological framework to estimate the influence of one region on another, while it also allows us to test our hypothesis on model structure and to decide which models are more plausible than others considering the data. However, little is known about optimal strategies for model search in DCM.

One major hindrance in this field is the high computational demand that parameter estimation poses. To use the algorithm for model search purposes, performance improvements are required. Some solutions, available for fMRI (Aponte et al., 2016) and EEG (Wang et al., 2013) data, use the graphical processing unit (GPU) to perform matrix operations applied frequently. These methods usually utilize an alternative parameter estimation procedure to complement their massively parallel nature. We decided to implement the exact DCM12 algorithm from SPM to achieve the same estimation results of the posterior parameter densities as the original algorithm does. Using ReDCM with high-performance computing techniques we were also able to estimate all 10*65,536 models in acceptable time. We make the database of the entire computed model-space freely available for any research group to develop model search methods and to further investigate the properties of the DCM model-space. In this study, we separated the DCM model estimation from a model search to facilitate the development of search algorithms. Looking up results of a previously estimated model-space provides an efficient framework that developers of model search methods can exploit for testing and optimizing searching procedures.

Another crucial point of DCM is that Fe is only an approximation of the log model evidence. As it estimates the log-evidence of a model under a Laplace approximation, this measure depends on prior parameter densities that are chosen to ensure the VL algorithm to converge. Hence the prior parameters of a model are defined by the structure of the model, and careful consideration is needed when comparing models with different numbers of connection parameters (Penny et al., 2004). To address this problem, DCM minimizes the Kullback-Leibler divergence (Kullback and Leibler, 1951) between the prior density and the approximate posterior (Friston et al., 2007). This measure can be seen as the complexity of the model and is required to compare models with different structures. For searching models, our methods used Fe to compare models with different structures (and complexity), but we can also obtain useful information from a model's posterior connection parameter estimates. Here we demonstrated that model search algorithms can be improved by analyzing individual parameters of a model besides considering their free-energy.

However, finding the one model with the highest Fe may not be meaningful. Even if the best balance between model fit and complexity is found and lower complexity models are more preferred than dense model structures, we cannot be sure whether the models are overfitting the data or not. In practice, it is encouraged to form a hypothesis about a network feature, and split the model-space into two (or more) groups or families around the tested connectivity patterns. In this study we showed that topological model search methods can successfully identify patterns of the winning model family constructed by a priori hypothesis about network structure.

The model search algorithms we used to find the best model are based on iteratively changing the structure of the models to improve model fitting. This approach assumes that models close to the best one in Fe are also close in structure (or Hamming-distance in this case). This assumption holds to some extent, but it is still unclear how free-energy and Hamming-distance are distributed over an arbitrary dataset and how these characteristics are related to each other. We found that these methods can be applied to achieve useful models, although they are not always reliable. A limitation of using topological search algorithms, generally used for graph discovery, is the questionable generalizability for any input data or different model structures.

A method for *post-hoc* model selection, described in the work by Friston and Penny (2011), finds an optimized reduction for any base model using a greedy approach removing free parameters from the model. While it efficiently and quickly optimizes model evidence by reducing connection parameters to shrinkage priors, it strictly remains in the Bayesian framework without considering graph structural aspects of network modeling. As the optimized models can violate model-space restrictions we set, it should be used on subject-level data with care. In most cases it means that even those endogenous connections (i.e., self-connections) can be removed from the model that are still modulated by any experimental input. However, reenabling these parameters later for easier interpretation or to match model-space restrictions moves results from their local minima, reducing search efficiency compared to topological methods. In contrast, the linear model of group-level PEB can be efficiently reduced with BMR, while topological methods are limited to search variations among parameters for common group effects rather than all parameters for explanatory variables within the PEB framework. For this reason BMR on PEB models can be considered the ‘gold standard’ and currently recommended way for group-level hypothesis testing and network discovery method. To mitigate limitations for structural issues one could extend model search algorithms to search over PEB model parameters rather than model structures defined by first-level DCMs.

In summary, we characterized three graph theory based model search algorithms adapted for DCM and compared their efficiency based on free-energy difference and Hamming-distance relative to the best model in the model-space and the number of estimated models during search. We included the BMR-based *post-hoc* model selection in the comparison, and discussed advantages and disadvantages of different approaches. We found that topological algorithms often outperform analytic (BMR) methods while searching for the optimal model structure on the subject-level. Furthermore, each algorithm performs well in finding models that share properties described by the winning family in a family-wise model selection scheme. However, topological methods reveal their limitations in searching through nested PEB models, which confirms BMR to be the currently recommended way to optimize group-level models. To develop and test model search methods efficiently, we separated DCM computations from generating model alternatives, replacing model inversion with time efficient database lookups. We share model-space data used in this study, and the ReDCM R package, which reimplements deterministic bilinear DCM to support high-performance computing facilities. We hope that our work will help further studies of the DCM model-space and the ReDCM package will be a useful contribution for Bayesian inference in the field of neuroscience.

## Data Availability Statement

The generated database of parameter estimates and model search results supporting the conclusions of this article are available at https://aranyics.github.io/ReDCM/. The data table contains model description and DCM parameter estimation results for each model in the model-space. The current version of the ReDCM R package and source codes can be downloaded from the GitHub repository of the package: https://github.com/aranyics/ReDCM/releases. The implemented algorithm is based on DCM12 from the v6225 build of the SPM toolbox.

## Author Contributions

SA and ME took part in study conceptualization and development of model search methods. ReDCM software was developed by SA. GO managed hardware resources and data storage for model-space data. SA, MN, and ME contributed to writing the original manuscript and visualization. EB and ME was responsible for funding acquisition and project supervision. All authors revised and approved the submitted manuscript. All authors contributed to the article and approved the submitted version.

## Conflict of Interest

The authors declare that the research was conducted in the absence of any commercial or financial relationships that could be construed as a potential conflict of interest.
